# Conservative Management of Late-Onset Legg-Calvé-Perthes Disease in a 10-Year-Old Child: A Case Report

**DOI:** 10.31729/jnma.8954

**Published:** 2025-04-30

**Authors:** Rishi Ram Banjade, Sandesh Dhakal, Prithu Nepal, Niraj Kumar Sharma

**Affiliations:** 1 Kathmandu Medical College and Teaching Hospital, Sinamangal, Kathmandu, Nepal; 2 Department of Orthopaedics, Kathmandu Medical College, Sinamangal, Kathmandu, Nepal; 3 Department of Radiology, Kathmandu Medical College and Teaching Hospital, Sinamangal, Kathmandu, Nepal

**Keywords:** *avascular necrosis*, *femoral head*, *legg-calvé-perthes disease*, *osteonecrosis*

## Abstract

Legg-Calvé-Perthes disease is a childhood condition characterized by avascular necrosis of the femoral head, with a poorer prognosis in children over 8 years. This case report describes a 10-year-old boy presenting with persistent left thigh pain, limp, and limited hip motion following a fall 3 months earlier. Imaging revealed osteonecrosis of the left femoral head with degenerative changes. Despite surgical intervention being the standard for late-onset LCPD, the patient was managed conservatively using bilateral skin traction and a Petrie's cast due to financial constraints. Follow-up monthly for 6 months showed significant improvement, highlighting conservative treatment's potential in resource-limited settings.

## INTRODUCTION

Legg-Calvé-Perthes disease (LCPD) was first described by Thornton Legg, Jacques Calvé, and George Perthes.^1^ It is an idiopathic avascular necrosis of the femoral head, primarily affecting children aged 4-7 years. The severity varies from mild cases with no long-term effects to severe degenerative hip changes.^2^ Males are more commonly affected, with a male-to-female ratio of 4:1 to 5:1.^3^ Risk factors include familial history (10%), delayed bone age, HIV, thrombophilias, secondhand smoke exposure, low birth weight, and socioeconomic status. Disease incidence is lower in equatorial regions and higher in Northern Europe.^4^ Prognosis depends on age, femoral head involvement, and treatment. Children over 8 have poorer outcomes.^1^

## CASE REPORT

A 10-year-old male presented with persistent left thigh pain that began three months prior and had worsened over the past month. The patient also exhibited a limp. He had a history of a fall from approximately 10 feet three months earlier. Initially, the pain was mild but progressively intensified to moderate severity, without radiation, aggravated by movement, and relieved by rest. There was no associated fever, chronic steroid use, recent viral infections, blood dyscrasias, or previous surgeries.

The patient first sought care at a local hospital where symptomatic management was attempted; however, his symptoms persisted. He was subsequently referred to the Orthopaedics outpatient department of our Tertiary care center for further evaluation and management.

On presentation, the patient displayed an antalgic gait on the left side. The skin over the affected area was intact, with no increase in temperature or tenderness noted. Range of motion in the left hip was limited: flexion was 110 degrees, extension was 10 degrees, adduction was 10 degrees, abduction was 10 degrees, internal rotation was 10 degrees, and external rotation was 30 degrees. There was also a measurable difference in thigh circumference, with the left thigh measuring 28 cm and the right thigh 30 cm.

**Figure 1a:**
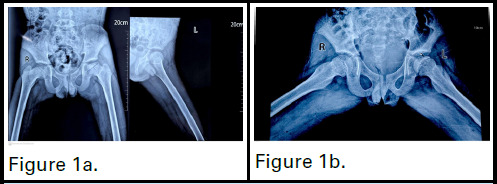
X-ray AP view of the hip showing loss of femoral contour on the acetabulum. Figure 1b. X-ray frog leg view of the Hip showing the femoral head over the acetabulum on abduction.

**Figure 2a:**
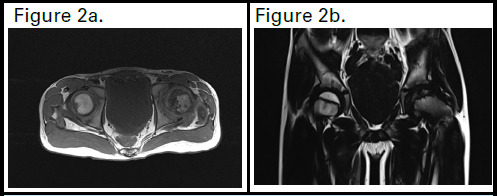
A sagittal section at the level of the femoral head showing necrosis of the femoral head. Fig 2b. shows an irregular contour of the femoral head over the acetabulum.

Initial plain radiographs revealed involvement of more than 25 % of epiphysis and lateral pillar height is reduced showing flattening of the left femoral head(Fig. 1a,1b). MRI scans (T1, T2, and proton density (PD)-weighted images with and without fat saturation) showed over 25% epiphyseal height loss in the left femoral head, on the lateral aspect, with reduced marrow signal intensity. A linear low-signal subchondral area suggested sclerosis and T2/PD cleft narrowing indicated subchondral collapse. Mild T2/PD high signal in the femoral epiphysis suggested edema. Findings were consistent with osteonecrosis and secondary degenerative change(Fig. 2a,2b). According to the Catterall classification, it is classified as Type II, where 25-50% of the epiphyseal region is affected by the lesion. In the Herring lateral pillar classification, it falls under Group B, indicating that there is less than 50% loss of height in the lateral pillar.

Although surgical treatment is recommended in the literature for late-onset Perthes disease, financial limitations prevented our patient from undergoing surgery, leading us to pursue a conservative management approach instead. The patient was managed conservatively with analgesics and bilateral skin traction for three weeks, followed by gradual abduction over 7 to 10 days to immobilize the joint. This was followed by the application of Petrie's cast. The patient was discharged with the Petrie's cast and was scheduled for monthly follow-ups, during which X-rays of the left thigh (AP and lateral views) were taken and reviewed.

**Figure 3a, 3b:**
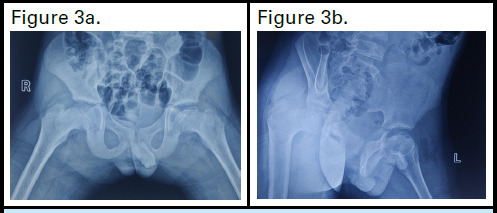
An AP and lateral view of the X-ray hip, showing improvement in the contour of the femoral head over the acetabulum after 4 months.

After four months of follow-up, the round contour of the femoral head was regained, as shown (Fig. 3a,3b). At this point, Petrie's cast was removed, and the patient was advised to begin physiotherapy with non-weight-bearing exercises. After one week, the patient was instructed to gradually transition to weight-bearing activities. Gradual improvement in the patient's condition was observed over 6 months period.

## DISCUSSION

Though we have limited information in terms of the predisposing factors for our patient's diagnosis, we could consider a fall from 10 feet height 3 months earlier to be the predisposing factor for a disruption of femoral head blood supply that led to LCPD. The most effective imaging modality for LCPD initial diagnosis and subsequent follow-up is still simple radiographs. In this method, the femoral head's dimensions and form are significant.^5^ Early in the disease, MRI differentiates osteonecrosis from other disorders by revealing bone infarction extent and the cartilaginous head and labrum anatomy.^6^ The treatment of Perthes disease focuses on preventing hip arthritis in adulthood by minimizing early femoral head deformation, ensuring early diagnosis to prevent further damage, and preserving the function of already affected hips.^7^

Several classification systems focus on the extent and location of the affected region to help predict the prognosis and guide treatment decisions. In 1971, Catterall developed a system to classify the condition based on the severity of the epiphyseal lesion, dividing it into four grades: Type I involves 0-25% of the epiphysis, Type II affects 25-50%, Type III impacts more than 50%, and Type IV encompasses 100% of the epiphyseal area.^8^ In 1992, Herring proposed a classification based on the lateral pillar height of the femoral head during the fragmentation stage. Patients are grouped as follows: Group A-lateral pillar intact; Group B-less than 50% height loss; and Group C-more than 50% height loss. This system aids early prediction of disease progression.^9^

In our case, the patient initially presented at Catterall Stage II or Herring Type B classification. After four months of abduction in a Petrie's cast, there was a notable improvement, with the classification progressing to modified Stulberg I.

Patients with hip pain or stiffness should rest for 5-7 days, often with NSAIDs for relief. If symptoms persist, hospitalization is needed for bilateral skin traction with gradual abduction for 7-10 days to immobilize the joint.^10^ To maintain femoral head congruency with the pelvis, patients with extensive damage may undergo procedures like innominate or femoral osteotomy. For children in Catterall groups, III/IV or over 8 years old, shelf arthroplasty is advised. The short-term goals are to preserve hip range of motion and femoral head containment, while the ultimate aim is to maintain hip congruency and sphericity. Despite several options, the optimal treatment for late-onset cases remains uncertain.^11^

Late-onset Legg-Calvé-Perthes disease has a severe course; most conservatively treated patients develop early degenerative arthritis regardless of femoral head involvement. Thus, aggressive treatment is recommended for this age group.^12^ In contrast, younger patients generally respond well to conservative or symptomatic treatment and tend to have better outcomes.^1^ In study done by Arkadar A, no statistical difference was found between surgical and conservative treatments for late-onset Legg-Calvé-Perthes disease (LCPD) but there was a trend toward better radiographic outcomes when varus derotational osteotomy (VDRO) was performed early in the disease process.^11^ Acetabular lateral shelf, Salter, Chiari, and triple osteotomies reposition or enlarge the acetabulum for better femoral head support. However, they do not reduce pressure or reshape the head, and no traditional treatment has significantly improved Perthes’ outcomes. The search for optimal treatment for late-onset Perthes’ disease continues.^13^

In our cases of late-onset Legg-Calvé-Perthes Disease (LCPD), financial issues necessitated a conservative treatment approach. Rather than forgoing treatment, patient's families opted for non-surgical management, a more cost-effective alternative to surgery, to achieve the best possible outcomes within the available resources. After a follow-up period of four months, we observed significant improvement in the contours of the femoral head, demonstrating the effectiveness of non-invasive methods such as abduction with Petrie's cast and physiotherapy. In study conducted by I. Epillito on conservative management of late-onset Legg-Calvé-Perthes disease, six out of ten patients achieved favorable functional outcomes. However, despite these positive functional results, early-onset osteoarthritis was frequently observed in this group.^12^ Similarly, in study done by Fulford GE in children with Perthes’ disease, two groups treated with bed rest and traction followed by either a weight-relieving caliper or proximal femoral varus osteotomy showed similar outcomes.^14^ These findings indicate that conservative management in the early stage of late-onset LCPD can be a feasible treatment option, maximizing outcomes within resource limitations and demonstrating the potential for favorable results without surgical intervention. However, given the limited case reports and the variability in disease progression among patients, further research with larger cohorts is necessary to establish the broader applicability and long-term effectiveness of this approach in managing late-onset LCPD.
